# Acute Intoxication following Dimethyltryptamine Ingestion

**DOI:** 10.1155/2018/3452691

**Published:** 2018-02-27

**Authors:** Matthew H. Bilhimer, Rachel F. Schult, Kristan V. Higgs, Timothy J. Wiegand, Rachel M. Gorodetsky, Nicole M. Acquisto

**Affiliations:** ^1^Salina Regional Health Center, 400 S Santa Fe Avenue, Salina, KS 67401, USA; ^2^University of Rochester Medical Center, 601 Elmwood Avenue, Rochester, NY 14642, USA; ^3^Owensboro Health Regional Hospital, 1201 Pleasant Valley Road, Owensboro, KY 42303, USA

## Abstract

Ayahuasca is a hallucinogenic tea that is most commonly comprised of the vine* Banisteriopsis caapi* alone or in combination with other plants such as* Psychotria viridis*. This concoction results in an orally active form of dimethyltryptamine (DMT), a hallucinogenic amine. Despite use in South America as a medicinal agent and component in religious ceremonies, interest in its recreational use and spiritual effects has led to increased use in the United States. We describe a unique case following ingestion of ayahuasca tea in a patient with history of schizophrenia resulting in personal injury and property damage. A review of ayahuasca toxicity and evaluation of serious adverse effects is also presented.

## 1. Introduction

Ayahuasca is a hallucinogenic tea that is most commonly comprised of the vine* Banisteriopsis caapi* alone or in combination with other plants such as* Psychotria viridis* [1]. The hallucinogenic amine N,N-dimethyltryptamine (DMT), usually degraded by monoamine oxidases in the gut, is made orally active when the DMT containing leaves of* P. viridis* are consumed with the beta-carboline alkaloids present in* B. caapi* vines. Despite recorded use for centuries in South America as a medicinal agent and a component of religious ceremonies, interest in the psychedelic effects and perceived spiritual effects of ayahuasca has led to its increased use in the United States (US), especially among young adults [2–4]. We present a case of severe agitation following ayahuasca consumption with DMT confirmation.

## 2. Case Report

A 25-year-old male with history of schizophrenia, prior suicide attempt, and hallucinogen abuse presented to the emergency department (ED) under mental health arrest for altered mental status. Neighbors notified police after he had broken several windows in his home and was creating loud noise and disturbance. They arrived to find him in front of the house and fighting with a cat; he was bleeding from lacerations on his chest caused by the cat's claws. The patient was subdued with a conducted electrical weapon and restrained in the field for aggressive behavior. In the ED, the patient endorsed suicidal ideation and was reportedly amnestic to the events prior to hospitalization. On physical examination, his blood pressure was 116/71 mm Hg and his pulse was 88 beats per minute. Multiple abrasions were noted on his chest, feet, and hands in addition to a laceration on his forehead, which required sutures. He had mild clonus with 3+ patellar reflexes but was not overtly rigid. His pupils were dilated and he had flushed skin but no diaphoresis. The initial laboratory values were significant for creatine kinase (CK) of 895 IU/L, white blood cell count of 20 k/mm^3^, and urine immunoassay positive for amphetamines (confirmation negative; cocaine, benzodiazepines, tetrahydrocannabinol, and opiates also negative). A medical toxicology consult was requested and the patient admitted to drinking a hallucinogenic tea purchased from the Internet containing ayahuasca. He reported taking no prescription or over-the-counter medications. He was admitted for medical observation and given intravenous fluids. Over the course of the first hospital day his mental status returned to baseline. The CK peaked at 1499 IU/L after 36 hours. A reference lab analysis later showed greater than 2,000 ng/mL dimethyltryptamine (DMT) in his urine. The patient was medically cleared after a 24-hour observation period and then admitted to an inpatient psychiatry unit for suicidal statements before eventually being discharged home on hospital day four with primary care provider follow-up.

## 3. Discussion

The hallucinogenic effect derived from ayahuasca ingestion results from an interaction between compounds present in each plant. The beta-carboline alkaloids harmine, tetrahydroharmine, and harmaline found in the vines of* B. caapi* possess the ability to inhibit monoamine-oxidase subtype-A (MAO-A) enzymes, which are found in the gut and liver. An additional alkaloid, DMT, is a tryptamine derivative similar to serotonin that is found in the leaves of* P. viridis*, but not* B. caapi *[5]. When administered via the inhalation or injection routes, DMT displays potent hallucinogenic effects. However, oral consumption of DMT results in no systemic activity due to degradative action of MAO-A enzymes in the gut [6]. Therefore, the hallucinogenic and psychedelic effect of ayahuasca is considered a result of DMT made orally active through the MAO-A inhibitor activity of* B. caapi *components.

The effects of ayahuasca use are well described and include visual imagery, alterations in auditory perception, increased thought processing leading to new associations, and often a state of introspection [5]. In healthy volunteers, the cardiovascular effects of ayahuasca use are generally considered mild to moderate as demonstrated by increases in systolic blood pressure and heart rate [7, 8]. Higher doses demonstrate proportional changes in described effects accompanied by nonsignificant increases in systolic blood pressure. There may also be a tolerance that occurs and results in less significant changes in cardiovascular effects with repeated dosing [7]. The most common side effects are nausea and vomiting; however, this consequence is sometimes desired by users that desire to elicit a catharsis [5].

There are few reports of serious, life-threatening reactions occurring with DMT or ayahuasca use. A few case reports suggest that various ayahuasca formulations have played a role in isolated fatalities; however, the lack of key information such as past medical history, blood analysis for beta-carboline alkaloids, brew composition, and dose ingested limits the ability to draw definitive associations [9, 10]. Television and magazine reports have also described significant, sometimes fatal, adverse reactions yet such accounts do not provide forensic evidence such as autopsy findings and chemical composition of the ingested compound [11].

There are concerns for drug-drug interactions between ayahuasca and monoamine-oxidase inhibitor and serotonergic medications that may result in serotonin toxicity. This phenomenon has not been observed commonly in people taking antidepressants with concomitant ayahuasca; however, case reports exist [12]. Callaway and Grob detailed a case of suspected serotonin toxicity in a 36-year old male receiving fluoxetine 20 mg orally each morning after drinking ayahuasca tea. One hour after consuming the brew, he collapsed and self-reported diaphoresis, tremors, and severe nausea and vomiting. His experience was accompanied by significant mental anguish and despair. Despite being physically incapacitated for several hours, he quickly became asymptomatic and was without long-term adverse outcomes [13]. This report is similar to our patient in that he was psychologically altered, although our patient did not take any medications at baseline making his presentation due to a drug-drug interaction unlikely.

The incidence of psychosis following ayahuasca tea alone appears unclear. One study suggested that the event is rare, occurring in less than one percent of ingestions in a report detailing approximately 25 psychotic episodes in 25,000 ingestions over a five-year period [14, 15]. In contrast, 34% of calls to poison control centers in the US for ayahuasca ingestion between 2005 and 2015 described agitation [3]. It is difficult to assess the significance of these episodes considering there are few published case reports. It has been hypothesized that patients with underlying psychiatric illness are more prone to psychotic effects. Szmulewicz et al. discussed a case of a 30-year-old man that experienced a manic episode after an ayahuasca ritual. At the time of the ingestion, there was no diagnosis of bipolar disorder but he was able to describe at least one hypomaniac episode in addition to his father having suffered from bipolar disorder type I [16]. However, despite such reports, there is also evidence suggesting ayahuasca may have clinical benefit in treating anxiety, treatment resistant-depression, and drug and alcohol dependence [17, 18]. Regardless of this reported benefit, our case provides support for an association between mental illness and psychotic behavior in that our patient had known schizophrenia and presented with acute agitation.

An additional component of our case that deserves attention is the act of violence that resulted in property damage and personal injury. There are no reports in the literature with confirmed ayahuasca consumption and associated violence or bodily injury. One case describes a man stabbed and killed by another during an ayahuasca ceremony; however, no forensic data (blood analysis or toxicology screen) was available for confirmation [19]. To our knowledge, our patient is the first case of confirmed DMT poisoning accompanied by significant psychosis resulting in personal injury and property damage.

Our report is not without limitations. First, we did not run quantitative blood test to analyze serum concentrations of DMT; however, the patient did not alert the treatment team to the presence of the ayahuasca tea for several hours after presentation. By this time, the patient had already convalesced and, given its short duration of action, a substantial proportion of DMT would have already been eliminated in his urine. We do not know the composition of the product nor the amount ingested but would likely never know, as the patient was reportedly amnestic to the events. It is unclear how much time had elapsed from the ingestion, psychosis, arrest, and subsequent evaluation in the ED. Although his behavior and vital signs during his evaluation in the ED were appropriate, his initial presentation in the field was significant enough for law enforcement officers to be concerned for their own safety and physically restrain him. His past history of schizophrenia may have also played a role in this episode, the degree to which the hallucinogenic tea consumed may have exacerbated this underlying medical condition is unknown. Finally, an additional toxin may have contributed to his presentation but his urine drug screen was positive only for amphetamines with final reference lab identification of DMT. The possibility exists that the amphetamine result was a false positive due to cross reactivity with DMT and lack of confirmatory testing but the presence of an additional undetected toxin cannot be ruled out.

## 4. Conclusion

Ritualistic use of ayahuasca is most prevalent in South American rainforests but is gaining popularity in the US. It is associated with visual imagery and a sense of introspection. Generally, this intoxicant combination is well tolerated with gastrointestinal upset and mild cardiovascular effects most commonly reported. Reports of serious, life-threatening reactions or acts of violence are rare and often without close, scientific critique and forensic validation. We present a case of significant psychosis resulting in property damage and personal injury after ingestion of a tea containing DMT. Considering the purported benefits of ayahuasca ingestion and its increasing popularity as a psychedelic substance, it is necessary to be aware of its toxicological effects so as to ensure consumers and health care providers are adequately informed.
